# Strategy to Establish Embryo-Derived Pluripotent Stem Cells in Cattle

**DOI:** 10.3390/ijms22095011

**Published:** 2021-05-09

**Authors:** Daehwan Kim, Sangho Roh

**Affiliations:** 1 Department of Bioengineering and QB3, UC Berkeley, Berkeley, CA 94720, USA; dhpark@berkely.edu; 2 Cellular Reprogramming and Embryo Biotechnology Laboratory, Dental Research Institute, Seoul National University School of Dentistry, Seoul 08826, Korea

**Keywords:** bovine, embryonic stem cells, induced pluripotent stem cells, extended pluripotent stem cells

## Abstract

Stem cell research is essential not only for the research and treatment of human diseases, but also for the genetic preservation and improvement of animals. Since embryonic stem cells (ESCs) were established in mice, substantial efforts have been made to establish true ESCs in many species. Although various culture conditions were used to establish ESCs in cattle, the capturing of true bovine ESCs (bESCs) has not been achieved. In this review, the difficulty of establishing bESCs with various culture conditions is described, and the characteristics of proprietary induced pluripotent stem cells and extended pluripotent stem cells are introduced. We conclude with a suggestion of a strategy for establishing true bESCs.

## 1. Introduction

Somatic cell nuclear transfer (SCNT) is a technique for generating embryos with genomic information identical to that of donor cells. This technique was first reported using frog somatic cells in 1962, and the first successful cloning in mammals was achieved by SCNT in 1997, creating Dolly the sheep [1,2]. Over the last five decades, clones have been successfully produced in many species, including the non-human primate species of macaque monkeys [3,4,5,6,7,8].

Although there are minor differences, the basic concept of SCNT is similar between species [9]. The first step of SCNT is the removal of the haploid chromosomes, including the meiotic spindle complex from a metaphase II stage oocyte, called enucleation. Then, a diploid donor cell is transferred into an enucleated oocyte, and electrical cell fusion is carried out to expose the nucleus to the ooplasm. Finally, artificial activation is performed by electric pulses or chemical stimulation, resulting in induction of the early development stage of the embryo.

In bovine, SCNT embryos and cloned offspring have also been reported [10,11,12]. Subsequently, the SCNT technique was used to make transgenic cattle. SCNT technology with transgenic cells providing a donor nucleus is one of the most efficient techniques for generating transgenic animals. Initially, the advantage of SCNT clones with transgenic cells is to provide increasingly good livestock products for human consumption.

Transgenic cattle are also considered as bioreactors for recombinant proteins. Many attempts have been made to generate cloned cows producing different human proteins, such as α-lactalbumin [13], lysozyme [14], granulocyte colony-stimulating factor [15], and lactoferrin [16]. Although the production efficiency is still low, many researchers have recently succeeded in producing higher concentrations (up to 13.6 g/L) of human proteins from cloned cows [17]. In addition, recombinant human myelin basic protein produced by transgenic cows shows a protective effect as a vaccine against multiple sclerosis [18]. These results show the potential for various commercial applications of proteins produced by transgenic cows.

Since stem cell research has expanded, SCNT technology has entered a new era. Embryonic stem cells (ESCs) are derived from the inner cell mass (ICM) of the blastocyst, the pre-implantation embryo, and they have two distinct properties: unlimited self-renewal and the ability to differentiate into all kinds of cells.

Since ESCs were established in mice [19], several ESCs have been reported in various animals, including humans [20,21,22]. Two different types of representative ESC have now been identified, naïve and primed states, reflecting the cellular characteristics of pre- and post-implantation embryos, respectively [23]. Although these two states present very similar features, they also differ from each other in terms of some features, including their morphology, dependent signals for maintaining pluripotency, and their contribution to chimera formation [24]. In large animals such as cattle, substantial efforts have been made to establish ESCs. However, it is still challenging to establish genuine ESCs, which might be related to species-specific characteristics of their reproduction and development.

In this review, we focus on ESCs and other pluripotent stem cells (PSCs) in bovine species, along with their limitations. In addition, we present new insights to establish authentic bovine ESCs (bESCs) and SCNT-bESCs.

## 2. Overview of Pluripotent Stem Cells

Since two types of PSC in naïve and primed states have been characterized in mice [23], the naïve and primed states are considered key criteria for genuine PSCs. In a broad sense, naïve and primed PSCs share core characteristics of pluripotency. They show unlimited self-renewal and the ability to differentiate into three germ layers [19,24]. However, they are not the same. In general, mouse ESCs (mESCs) from pre-implanted embryos are considered to be in a naïve state, and they show distinct characteristics, including small dome-shaped morphology, DNA hypomethylation, and two activated X chromosomes in females. To maintain their pluripotency, mESCs rely on leukemia inhibitory factor (LIF) and bone morphogenetic protein (BMP4) signals [25]. Since they can be recolonized through single-cell passaging, chimeras can be produced and show germline transmission. On the other hand, epiblast stem cells (EpiSCs) from post-implanted mouse embryos are considered to be in a primed state [24]. These cells show unique characteristics, different from the naïve state, such as large and flattened shapes, DNA hypermethylation, and X inactivation status in females. Moreover, EpiSCs retain their pluripotency under basic fibroblast growth factor (bFGF) and transforming growth factor beta (TGF-β)-related Activin/Nodal signaling [20]. Compared with the naïve state, mouse EpiSCs lack single-cell proliferation ability and, thus, cannot form chimeras. The transcription of genes of primed PSCs also differs from that of their naïve counterparts. The core pluripotent genes, OCT4, SOX2, and KLF4, are expressed in both; however, in naïve mESCs, STELLA and REX1 are expressed, while FGF5, T, and LEFTY, which are generally associated with differentiation, are expressed only in EpiSCs. Human ESCs (hESCs) are considered to be in a primed state, showing flat morphology and retaining their pluripotency under bFGF and TGF-β signaling. However, hESCs are not identical to mEpiSCs. For example, the pattern of transcription differs markedly, including FGF5, E-CADHERIN, and NANOG [26]. Moreover, some naïve state markers are expressed in hESCs, including PRDM14 and REX1 [27]. These results show that there are differences among species even though they are in a similar pluripotent state, and it is necessary to consider the species specificity to understand the mechanisms of the maintenance of their pluripotency.

## 3. Bovine Embryonic Stem Cells

After ESCs were successfully established in mice and humans, authentic ESCs were generated in several species [21,22]. There have also been attempts to establish ESCs in large animals, including cattle (Table 1). However, it is still challenging to establish bESCs that meet the criteria of true ESCs.

Representative stem cells show two different morphologies: a dome shape for the naïve state and a flattened shape for the primed state. Although some reports have depicted that the morphology of putative bESCs is similar to that of mESCs or hESCs, most shapes of colonies from bovine embryonic cells are heterogeneous, incompact, and irregular with an ambiguous description [28,29,30]. It was also reported that putative bESCs contain trophectoderm (TE). These cells sometimes show unexpected cystic cavities [31,32,33]. Our group attempted to generate putative bESCs from in vitro production (IVP), parthenogenesis (PA), and SCNT embryos [32,34]. There were no major differences in morphology among them. However, these putative stem cells were morphologically different from conventional ESCs. The colony showed two different parts: a central multilayer (CMt) and a peripheral monolayer (PMn). Mainly, the CMt part existed inside a colony and the cells were small and compacted in a clump, whereas the PMn part existed near the boundary and contained large and flattened cells. Unlike PMn, the CMt part was recolonized as a new colony after passaging, suggesting that only the CMt part may include pluripotent cells. The unstable appearance of bESCs means that authentic PSCs have not yet been established.

Like other PSCs, pluripotency marker genes were shown to be strongly expressed in putative bESCs. In particular, it was reported that genes related to naïve state are expressed in putative bESCs [32]. However, the expression of CDX2, a caudal-type homeodomain TF, was also reported in many putative bESCs [31,32,33]. Generally, Cdx2 expression was shown to be inhibited by Oct4 in mESCs [35]. Upon the overexpression of Cdx2, ESCs can differentiate into the trophoblast lineage, which does not happen spontaneously [35,36]. Although this heterogeneous population in bovine species cannot be fully explained, several reports that can help our understanding based on the knowledge of embryogenesis have been published. In early mouse development, the first cell fate is committed after the compacted morula stage, resulting in segregation into two different cell lineages: ICM, which will be the embryo, and TE, which will be the placenta. Cdx2 is an essential gene for TE fate determination, which acts by inhibiting Oct4 and triggering genes required for placental differentiation [37]. In addition, the Cdx2 mutant was shown to exhibit failure of embryo implantation [35]. In cattle, several studies on the transcriptome of early-stage embryos have been performed to find clues about the mechanism of early development. According to previous studies, TE-related markers, CDX2, TEAD4, and GATA4, were expressed in the ICM of bovine blastocyst, but not in the murine counterpart [38,39]. Moreover, there is little difference in CDX2 expression between ICM and TE in bovine blastocysts [33,37,40,41]. Furthermore, bovine embryos exhibit delayed implantation compared with murine and human embryos [33,42]. These findings suggest that this delayed expression of CDX2 may contribute to retard the first cell fate decision, resulting in an incomplete blastocyst stage and allowing TE to partially remain pluripotent. A report has described that cells derived from TE can contribute to ICM when injected into the early embryo [43]. This uncommitted TE may be considered to be able to easily contaminate ICM-derived cells during in vitro cultivation, resulting in some putative bESCs, including TE-derived cells.

Since the expression of CDX2 may impede bESCs in exhibiting true pluripotency, a CDX2-knockdown embryo (CDX2-KD) model was studied [33]. To understand the role of CDX2 in bovine pluripotency, SCNT embryos with CDX2-KD were generated. Unlike in mice, the CDX2-KD embryo not only formed an extended blastocyst stage, but had no significant effect on pluripotency marker gene expression. Embryo-derived stem cells were established from CDX2-KD and showed the expression of various pluripotency markers as well as in vitro and in vivo differentiation capacity. In terms of the shape, a human-like flattened shape was shown. Interestingly, despite the inhibition of CDX2, the expression of TE differentiation markers was increased when they differentiated spontaneously. This implies that the characteristics of putative bESCs differ from those of hESCs and mESCs.

To understand pluripotency, several transcriptome studies have also been conducted in cultured cells from bovine blastocysts. With various blastocyst-derived cells, microarray results suggested signaling pathways for bovine pluripotency [44]. Three different putative bESCs from IVP, NT, and PA embryos were used, with the findings revealing common increases in TGF-β, Wnt, and LIF signaling pathways related to pluripotency, implying that these pathways may be pivotal for capturing authentic pluripotency in bovines. Epigenetic patterns have also been reported to understand bovine pluripotency [45]. This paper confirmed that 62% of the H3K4me3-only bovine gene is shared by hESCs and mESCs. Interestingly, the patterns of H3K4me3 and bivalent genes were similar to those of hESCs rather than mESCs. In addition, with RNA-seq, the expression of primed-specific genes was found to be higher than that of naïve genes. These results suggest that bovine pluripotency may be close to that of hESCs. These reports indicate that transcriptome analysis might be useful for understanding bovine pluripotency, leading to the establishment of true ESCs.

## 4. Small Molecules for Capturing Bovine Pluripotency

In the initial culture system for bESCs, medium containing FBS, LIF, and bFGF that mimics mESCs or hESCs has been widely used (Table 1). However, these cells still could not meet the criteria of true or genuine ESCs. These differences suggest that the culture system to induce and/or maintain bESCs would differ from that of conventional PSCs.

Recently, it has been reported that small molecules can support the maintenance of mESCs and hESCs without differentiation [51]. In particular, this approach solved the difficulties in capturing ESCs from other species [51,52]. Rat was considered a species for which pluripotency could not be captured using conventional methods. However, true rat ESCs (rESCs) have been successfully established by inhibitors targeting the FGF receptor, MEK, and GSK3 [52]. rESCs with the treatment of small molecules show small dome-shaped clumps like mESCs and can differentiate both in vitro and in vivo. Moreover, chimeric rats were generated when rESCs were introduced into rat blastocysts.

In PSCs, the Wnt signal has been highlighted as one of the core pathways to support pluripotency. Although the mechanism of Wnt signaling in stem cells is still controversial [53,54], Wnt/β-catenin signaling is considered the key pathway for maintaining PSCs in not only mice but also humans [55,56,57]. Moreover, it was confirmed that the induction of naïve hPSCs requires Wnt signaling [58,59]. The Wnt pathway may contribute to regulating the levels of OCT4, NANOG, and SOX2 to support stemness of PSCs [56,57] and promotes somatic cell reprogramming [60,61]. These results are thought to be essential for the effect of Wnt signaling to maintain pluripotency regardless of stem cell status or species. Wnt signaling may play an important role in contributing to the regulation of pluripotency in cattle. A transcriptomic study in bovine embryos demonstrated that Wnt signaling enhances pluripotency [39]. Moreover, several publications have reported on small molecules that can capture putative bESCs [32,50].

Like mice, dual inhibitors were used to establish putative bESCs [50]. They were demonstrated to have a morphology similar to that of mESCs and confirmed to have naïve state characteristics, including naïve gene expression and silenced X chromosomes. However, long-term cultivation of these cells failed, and they showed cystic EBs that were similar to those of a trophectoderm lineage.

To improve the establishment of bESCs, three inhibitors (3i), PD18435, SU5402, and CHIR99021, were also used [32]. The cells with 3i could undergo long-term cultivation of more than 50 passages, and their in vitro and in vivo differentiation was confirmed. Although TE-specific genes such as *CDX2* were still expressed, the 3i system appeared to support the expression of not only core genes, including OCT4 and NANOG, but also naïve-related genes including REX-1, KLF4, and NROB1, but not genes associated with a primed state, namely, T and LEFTY2 [20,32]. Moreover, cell populations positive for only OCT4 or NANOG without CDX2 expression were present in the 3i system. Although the colonies still comprised a heterogeneous population, this study showed that the 3i system might play a role in supporting intact pluripotency and suppressing TE differentiation of the cells that also express CDX2 in the area of CMt.

Recently, primed bESCs were reported, with bFGF and IWR1 being used to retain their pluripotency [45]. The primed bESCs expressed many genes related to pluripotency, and the expression pattern resembled one that is closer to a primed state than a naïve state. Moreover, CDX2 and GATA6 were not detected in the primed cells. Like other stem cells, the abilities of primed bESCs to differentiate into three germ layers in vitro and in vivo were confirmed. However, the primed bESCs showed irregular morphologies with an unclear boundary. It is also necessary to verify whether the culture conditions would support other types of bovine PSC.

Interestingly, unlike in humans and mice, several studies have reported that TE marker expression was observed in putative bESCs. Moreover, OCT4 is expressed in CDX2-positive TE and can contribute to ICM, suggesting that the fate of TE cells may not be determined entirely during the blastocyst stage, and it is assumed that the cells still have bipotent characteristics. Therefore, it is thought that the culture conditions of totipotent or bipotent stem cells may help to maintain bovine pluripotency, rather than the standardized culture conditions for humans and mice.

## 5. Other Pluripotent Stem Cells in Cattle

Induced pluripotent stem cells (iPSCs) are made from differentiated cells by reprogramming using Yamanaka’s four factors [62]. Like ESCs, iPSCs proliferate indefinitely and can differentiate into all cells of the body. In mice, iPSCs also contributed to generating chimeras when they were injected in the pre-implantation stage, implying that the potential of iPSCs is similar to that of ESCs. Upon various comparisons between ESCs and iPSCs, it has been found that the dependence mechanisms for gene expression and maintenance of pluripotency, as well as the epigenetic patterns, are very similar to each other [63]. Since there are ethical problems associated with ESCs, iPSCs are considered a substitute [64]. Interestingly, both cells can be cultured in the same culture medium [62,65]. However, there is still a difference from reprogramming through oocyte-derived factors, so additional research is needed to ensure safe use [66,67].

It has been reported that iPSCs can be established not only in humans and mice but also in several other animals [62,68,69,70,71]. Interestingly, the number of species for which iPSCs has be established is greater than the number of species for which ESCs can be established. This suggests that the mechanism of pluripotency regulation may differ among species in the early stages of development. As a result, it is difficult to find the timing to capture optimal pluripotency. However, once stable pluripotency is acquired, it can be assumed that the control mechanisms are similar among species. For this reason, it is estimated that the establishment of iPSCs may be easier than that of ESCs.

Many efforts have also been made to generate iPSCs in livestock. To capture bovine iPSCs (biPSCs) in vitro, various culture conditions have been suggested through well-established mechanistic studies in humans and mice (Table 2). Although complete culture conditions have not been established, biPSCs have also been reported, having the morphology of a dome shape similar to that of mESCs. They are considered to be cells in a naïve state depending on the LIF signal (Table 2). However, they still showed unstable states and limited proliferation [72,73]. In addition, for biPSCs, chimera production still needs to be verified, and there is a need to show that bESCs and biPSCs may be able to maintain their pluripotency through the same medium.

Recently, extended pluripotent stem cells (EPSCs) were demonstrated in humans and mice [74]. These cells can contribute to not only the embryonic lineage but also extraembryonic lineages such as totipotent stem cells. The first culture system of EPSCs in mice consisted of hLIF, CHIR99021, (S)-(+)-dimethindene maleate, and minocycline hydrochloride. These cells were able to undergo simple cultivation in culture medium without any forced gene expression. A single mouse EPSC (mEPSC) can contribute to both embryonic and extraembryonic lineages in vivo.

Moreover, bovine EPSCs (bEPSCs) were captured under the same conditions [75]. Like mEPSCs and human EPSCs (hEPSCs), a single bEPSC could differentiate into both cell fates, embryonic and extraembryonic lineages, when injected into the mouse embryo. However, the EPSC culture conditions did not appear sufficient to maintain expanded pluripotency in bovine species because the culture system was unable to capture the pluripotency from bovine embryonic cells. Moreover, the morphology of these cells was similar to that in a primed state, with a large flattened shape. Interestingly, bEPSCs were able to contribute to both lineages when they were introduced into embryos. However, these cells can only be obtained from iPSCs and not from embryos. There are several possible explanations for this. One is the differences in developmental patterns among species; to understand these better, additional studies of the characteristics of the cells at the embryonic stage are required. Although these culture conditions are not perfect for establishing bESCs, new possibilities are available for capturing the pluripotency of bovine (stem) cells. In addition, one study [75] provided new insights into the similarities and differences in establishing pluripotency between species. In particular, the mechanism of bovine pluripotency would be different from other species, and it may be closer to those of EPSCs contributing to both lineages than those of conventional PSCs.

Another cocktail was shown to be able to support mEPSCs [76]. Six inhibitors plus hLIF were used for TE/ICM segregation by targeting MAPKs, Src, and Wnt/Hippo/TNKS1/2. In this paper, EPSCs were generated from a blastomere, which is still at an uncommitted stage. These cells expressed pluripotency genes that were similar to conventional ESCs, but EPSCs can contribute to not only the ICM but also the TE. Even so, conventional ESCs and iPSCs were able to meet the expanded potential when cultured in the EPSC culture conditions.

**Figure 1:**
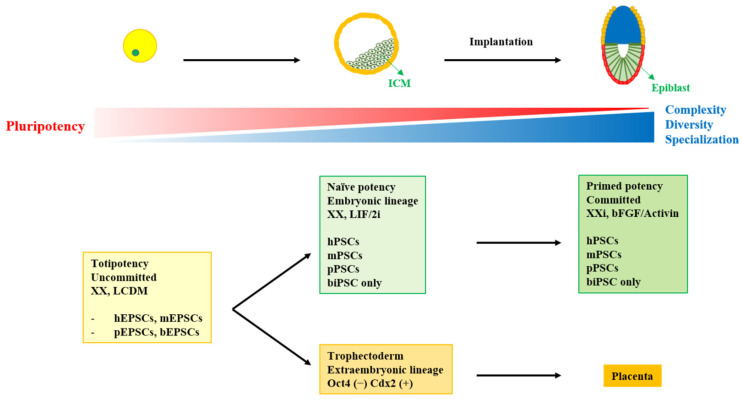
The relationship between developmental stages and pluripotency states. ICM: inner cell mass; X: active X chromosome; Xi: inactive X chromosome; LIF: leukemia inhibitory factor; 2i: a medium consisting GSK3 and Mek1/2 inhibitors; bFGF: basic fibroblast growth factor; LCDM; a medium consisting of LIF, CHIR99021, (S)-(+)-dimethindene maleate, and minocycline hydrochloride; EPSC: extended pluripotent stem cells; PSC: pluripotent stem cells; iPSC: induced pluripotent stem cells; h: human; m: mouse; p: pig; b: bovine.

Pigs used to be a species in which it was difficult to establish genuine PSCs. However, porcine EPSCs (pEPSCs) have been established using the same EPSC culture conditions as used in mice [77]. The pEPSCs can be generated from the porcine embryo, implying that the culture system could support the maintaining of the pluripotency of porcine ESCs. Interestingly, porcine iPSCs also exhibit the same potential in the same medium. Both cells in the same culture conditions met all of the criteria of true PSCs. In addition, these cells were able to contribute to not only the embryonic lineage but also the extraembryonic lineage. Interestingly, the EPSC culture is equally applicable to humans and mice [76,77]. It is considered that the culture conditions can overcome the differences among species (Figure 1). It is also suggested that bEPSCs could be established from bovine embryos with the six small molecules. These results are expected to have a significant impact both academically and commercially.

## 6. Applications and Future Perspectives

**Figure 2:**
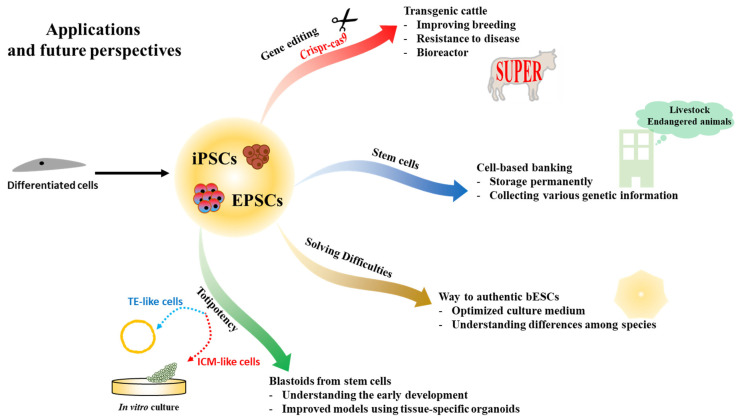
Applications and future perspectives of pluripotent stem cells in livestock. ICM: inner cell mass; TE: trophectoderm; EPSC: extended pluripotent stem cells; bESC: bovine embryonic stem cells; iPSC: induced pluripotent stem cells.

Capturing ESCs in large animals is still a challenge. Recently, EPSCs have been established using embryo or somatic cell reprogramming in pigs and cattle [75,77]. However, there are still gaps among pluripotent states. For example, it is possible to generate iPSCs in naïve and primed states from differentiated cells, but ESCs cannot be derived from embryos directly, even in the culture conditions of biPSCs. Bridging these gaps may provide important clues to help to establish and deeply understand the PSCs of various species, including bovine. However, the recently established EPSCs are expected to change the field not only from a research perspective but also in the livestock industry (Figure 2).

### 6.1. Transgenic Cattle by Genetic Editing

Initial genetic manipulation was performed in mESCs using various methods, including homologous recombination (reviewed in [85]). This technique was of great help in studying the functions of various genes with a gain/loss strategy. However, in general, this technique is difficult in cells with weak proliferative capacity, including fully differentiated cells, due to its low efficiency. Unlike differentiated cells, stem cells can proliferate eternally. Moreover, naïve PSCs and EPSCs show the vigorous ability of single-cell proliferation. For this reason, this low-efficiency hurdle can be overcome through robust proliferation.

In domestic animals, including bovine, studying the function of genes is not the priority. Instead, the goal of establishing transgenic animals in livestock is to promote economically important traits, such as improving breeding and fattening, and strengthening resistance to livestock diseases, including infectious ones. In addition, dairy cows are expected to act as bioreactors capable of producing specific proteins.

Genetically engineered stem cells can be employed to directly produce transgenic animals in livestock. Previously, breeding improvement depended on phenotypes, and it took a lot of time to improve and confirm traits through several generations. However, recently, stem cell technology with gene editing has allowed the production of transgenic animals more rapidly than before [86]. In particular, genetically modified animals can be generated in the first generation when stem cells are introduced into tetraploid embryos or used as donors for SCNT [87].

Germline transmission is considered to be essential in maintaining a new transgenic model [9,25]. Regarding this, genetically modified embryos in which endogenous germ cells are ablated during development, thereby producing a sterile animal, were used to generate chimeras that allowed 100% germline transmission [88]. Since indispensable problems such as low development and low fertility still remain in these assisted reproductive techniques, a rational approach is to mimic the production of functional germ cells and the natural fertilization process. Although several reports on this issue have been published, additional studies are needed to stably produce them in various species [89].

### 6.2. Cell-Based Banking in Animals

For decades, sperm, oocytes, and in vitro-fertilized embryos have been frozen for the long-term preservation of genetic traits, but this strategy showed low recovery efficiency after thawing due to physical damage that occurs during freezing [90]. Although differentiated cells, including fibroblasts, which are easier to obtain than reproductive cells, could be used as a donor and to preserve genetic information, SCNT embryos with these cells have low efficiency and a high level of abnormalities during the developmental process [91,92]. Recently, iPSCs have attracted attention as a new approach for storing genetic information [93]. This concept could also be applied to the livestock industry. Although true bESCs have not yet been established, iPSCs have been reported and they seem to be reproducible (Table 2). It is estimated that genetically engineered iPSCs may be stored permanently for various purposes based on this technology, such as for a cell-banking system [92,93]. With the data from this system, it is also considered possible to produce transgenic animals suitable for specific purposes, like immune-enhanced animals.

These techniques apply not only to livestock but also to endangered animals. In some endangered animals, iPSCs have already been established for the permanent conservation of genetic information. It is assumed that establishing iPSCs using various cells from animals that are endangered due to environmental changes could contribute to maintaining biodiversity [94,95].

### 6.3. Difficulties in Generating Bovine Embryonic Stem Cells

To date, iPSCs have been established in a greater variety of species than ESCs. Why is it difficult to establish ESCs? Recent reports [75,76,77] have proposed several suggestions to explain this (Figure 1).

One possible explanation is that the optimal time when pluripotency is sufficiently expressed in the developmental stage has yet to be determined. Since the stage of development differs from one species to the next and the stage-specific gene expression pattern is also different, finding the right time in early development will be important in establishing true bESCs. A second explanation is that there is still a need to analyze the gene expression of cells from pre-implanted embryos. This transcriptome may help to understand the mechanisms related to bovine pluripotency and contribute to establishing suitable in vitro culture conditions for bESCs. From the knowledge obtained so far, stem cell culture conditions appear to reflect the potential for pluripotency. To date, several studies have been conducted on transcriptome analysis of cells from early developmental embryos in bovine and suggested strongly related pathways such as Wnt signaling [38,39]. Therefore, it is suggested that unique culture conditions may be uncovered when the developmental clue is accurately connected.

Although true ESCs were not completely achieved, EPSCs from bovine and porcine species are considered a substitute for PSCs. These cells can meet the criteria of true PSCs and contribute to extraembryonic tissues. Additional research in this field is needed, but it is estimated that EPSCs can contribute to generating transgenic livestock.

At this point, a particularly interesting issue is how EPSCs can be established even in species where it is difficult to establish PSCs. Embryologically, the primed state is similar to the post-implantation stage and the naïve state is close to the pre-implantation stage [24,25], implying that the naïve state may have higher potential than the primed state. In this regard, EPSCs could be considered as a blastomere that can differentiate into both embryonic and extraembryonic lineages, so EPSCs may be expected to have higher pluripotency than PSCs. It is well known that the early stages of development in mammals are similar [96,97]. This suggests that there is low diversity among species and minimal differences of relevant mechanisms among uncommitted cells in the early developmental stages. In other words, the mechanisms maintaining cells or the blastomere are considered to be more similar than those in the first committed cells, ICM, and epiblast. For this reason, unlike in naïve and primed states, EPSCs are estimated to be less affected by species specificity. Another speculation is that features such as those of EPSCs may be common in bovine. Several reports have shown that biPSCs were able to differentiate into embryonic as well as extraembryonic lineages [75,98]. These results differ from the previously known features of conventional PSCs. Additional research is needed on these results, but this study provides a new perspective and insight on the standards of bovine stem cells.

### 6.4. Blastoids from Stem Cells

Recent studies have confirmed the ability of ESCs and EPSCs to generate early embryo-like structures called blastoids [99,100,101,102]. These blastoids showed similar transcriptional patterns to early developmental cells, and it is difficult to identify morphological differences between blastoids and blastocysts. Blastoids can be derived from ESCs and EPSCs with extraembryonic cells [100,101] or EPSCs alone [102]. In many cases, studies of ESCs or embryonic development encounter ethical limitations, especially in humans [64]. Although technologies with EPSCs and iPSCs are still in their infancy, blastoids from them may help to avoid some ethical issues. In addition, it is considered that blastoids may provide impeccable stories about early development, leading to an understanding of cell fate commitment and organogenesis. This information is also useful for establishing drug screening systems and for disease modeling. There are many limitations when studying rare human diseases, but it is believed that these problems could be overcome. Several recent studies have reported functional screening for treating diseases related to specific organs through organoids [103,104,105,106]. Moreover, these new technologies are becoming faster and more automated, including in differentiation, processing, imaging, and analysis [103].

However, there are still many hurdles to overcome in the early studies of blastoids. There are mutations and cell instability that occur in the artificial process of generating and culturing stem cells. In addition, the established stem cells still have the epigenetic memory and methylation status of the donor cells, which influence cell differentiation and fate determination [44,107,108]. Although gene editing, including CRISPR technology and epigenetic modifiers, are generally utilized, many unknown issues still remain, including side effects such as off-targeting. Moreover, all studies of blastoids have indicated that the embryonic process cannot be fully mimicked, so more research on their characteristics and potential is needed [99,100,101,102].

## 7. Conclusions

In this review, we introduced the characteristics of bovine-derived pluripotent stem cells of various origins. We also described the difference between putative bESCs and conventional true ESCs. Based on these results, we discussed how difficult it is to establish true bESCs. Differences in mechanisms for maintaining pluripotency in ESCs and iPSCs were evident among species. However, the boundaries of the pathways that maintain the potential of EPSCs appear to obscure the differences between species, so that they share culture conditions [74,75,76,77]. In the early stages of development, there are no major differences in the morphology of zygotes (single cells) among species, suggesting that the mechanism for pluripotency may be simple and similar between species. However, approaching the later stages of development, it seems that various committed cells with different morphologies can arise, and complex mechanisms to maintain each specialized cell are necessary (Figure 1). Therefore, it is estimated that the difference and complexity of pathways to pluripotency could be less for cells with higher levels of potential. This suggests that it is necessary to pay attention to species in which the establishment of true ESCs has failed. Bovine species comprise one option to consider when building a strategy to establish genuine bESCs, including SCNT-bESCs.

## Figures and Tables

**Table 1:** Various characteristics of bovine embryo-derived stem cells.

Medium	Morphology	Pluripotency	Differentiation	Special	Reference
FCS, heparin, LIF	mES-like cells	X	Epithelial, fibroblastic, neuron-type cells	With trophoblastic cell	[46]
FCS	Low cytoplasmic/nuclear ratio	X	in vitro differentiation	Trophecoderm-like cells	[28]
FBS, LIF	Monolayer cells	X	X	Tetraploid embryos test Contributing to liver, placenta, and hair roots in chimera	[47]
FBS	Small cytoplasmic/nuclear volume ratio	SSEA-1(+), SSEA-3(+), SSEA-4(+)	in vitro differentiation	Long term culture Cystic form observed like TE	[30]
FBS, LIF, EGF	Small cells compact colony	AP(+), SSEA-1(+), STAT3(+), OCT4(+) SSEA-3(−), SSEA-4(−)	in vitro differentiation	Chimeric test Contributing to both lineages	[48]
FCS, ITS, LIF, bFGF, EGF, 5-azacytidine	Heterogenetic morphology	REX1(+), OCT4(+), SSEA-4(+)	in vitro differentiation	5-azacytidine improved pluripotency and ability to differentiate	[29]
FBS, bFGF, SCF	Bubble-like or TE-like cell	OCT4(+), SSEA-1(+), SSEA4(+), AP(+)	in vitro differentiation	Stem cell factor (SCF), a cytokine that binds to the c-Kit receptor	[49]
PD0325901, CHIR99021	Flat-shaped	Naïve state markers(+) Primed state markers(−)	in vitro differentiation	GATA6 and CDX2 expression	[50]
bFGF, LIF, KSR	Dome-like (early passages) Flat-shaped (late passages)	OCT4(+), SOX2(+), NANOG(+), E-CAD(+), SSEA1(+), SSEA4(+)	in vitro and in vivo differentiation	TE related genes still expressed in CDX2-KD lines	[33]
PD18435, SU5402, CHIR99021	Heterogenetic morphology mixed with TE	Naïve state markers(+) Primed state markers (−)	in vitro and in vivo differentiation	OCT4 or Nanog positive cells without CDX2 negative	[32]
BSA, bFGF, IWR1	Flat-shaped	Primed state markers(+)	in vitro and in vivo differentiation	X	[45]

FCS: fetal calf serum; LIF: leukemia inhibitory factor; FBS: fetal bovine serum; EGF: Epidermal growth factor; ITS: insulin-transferrin-selenium; bFGF: basic fibroblast growth factor; SCF: stem cell factor; KSR: knockout serum replacement; BSA: bovine serum albumin; IWR1: a Wnt/β-catenin inhibitor.

**Table 2:** Various characteristics of bovine induced pluripotent stem cells.

Medium	Cell Source	Morphology	Reprogramming Factors *	Pluripotency	Differentiation	Reference
KSR, bFGF	MEF	Dome-like	bOSKMLN	AP, OCT4, SOX2, NANOG, SSEA1,4	in vitro and in vivo	[78]
PD0325901, CHIR99021, LIF	MEF	Dome-like	bOKSM	AP, OCT4, SOX2, KIF4, SSEA3, 4, TRA-1-60	in vitro and in vivo	[72]
FBS, bFGF, LIF	skin fibroblast	Dome-like	hOKMN	AP, OCT4, SOX2, KLF4, C-MYC, NANOG, SSEA1/4	in vitro and in vivo	[73]
FBS, bFGF, LIF	MEF	Flat-shaped	hO+pSKM	AP, OCT4, SOX2, KLF4, NANOG, SSEA1	in vitro and in vivo	[79]
LIF, FBS	testicular cells	Dome-like	hO	OCT4, SOX2, NANOG, SSEA1, SSEA4	in vitro and in vivo	[80]
KSR, bFGF, hLIF	BFF	Dome-like	hOSKM	OCT4, SSEA1, 3, 4, REX1	in vitro and in vivo	[81]
CHIR99021, PD0325901, Valproic acid	BFF	Dome-like	bOSKM	OCT4, SOX2, NANOG, KLF4, C-MYC, REX1	in vitro and in vivo	[82]
Bio, SC1, 5-AzaC	bAF	Dome-like	hOSKMN	OCT4, NANOG, SSEA-1, SSEA-4, TRA-1-60	in vitro and in vivo	[83]
FBS, LIF, bFGF	BFF	Dome-like	bOSKM	OCT4, NANOG, SOX2, SSEA1, SSEA4, AP	in vitro and in vivo	[84]
KSR, hLIF, CHIR99021, (S)-(+)-dimethindene maleate, and minocycline hydrochloride **	BFF	Dome-like	bOSKM	OCT4, SOX2, NANOG	in vitro and in vivo Interspecies chimeric embryo test Totipotent-like cells	[75]

* O-OCT4, S-SOX2, K-KLF4, M-CMYC, L-LIN28, N-NANOG. ** Culture conditions for pluripotent stem cells expanded from iPSCs. KSR: knockout serum replacement; bFGF: basic fibroblast growth factor; LIF: leukemia inhibitory factor; FBS: fetal bovine serum; hLIF: human leukemia inhibitory factor; 5-AzaC: 5-Azacytidine; SC1: extracellular signal-regulated kinase/mitogen-activated protein kinase inhibitor; Bio: 6-bromoindirubin-3′-oxime; MEF: mouse embryonic fibroblast; BFF: bovine fetal fibroblast; bAF: bovine adult fibroblast.

## Data Availability

Not applicable.
